# Prognostic value of serial (1,3)-β-d-glucan measurements in ICU patients with invasive candidiasis

**DOI:** 10.1186/s13054-024-05022-x

**Published:** 2024-07-12

**Authors:** Simone Carelli, Brunella Posteraro, Riccardo Torelli, Elena De Carolis, Maria Sole Vallecoccia, Rikardo Xhemalaj, Salvatore Lucio Cutuli, Eloisa Sofia Tanzarella, Antonio Maria Dell’Anna, Gianmarco Lombardi, Fabiola Cammarota, Alessandro Caroli, Domenico Luca Grieco, Maurizio Sanguinetti, Massimo Antonelli, Gennaro De Pascale

**Affiliations:** 1 Dipartimento di Scienze dell’Emergenza, Anestesiologiche e della Rianimazione, Fondazione Policlinico Universitario A. Gemelli IRCCS, Università Cattolica del Sacro Cuore, Largo A. Gemelli 8, 00168 Rome, Italy; 2 Dipartimento di Scienze Biotecnologiche di Base, Cliniche Intensivologiche e Perioperatorie, Università Cattolica del Sacro Cuore, Rome, Italy; 3 Dipartimento di Scienze Mediche e Chirurgiche, Fondazione Policlinico Universitario A. Gemelli IRCCS, Rome, Italy; 4 Dipartimento di Scienze di Laboratorio e Infettivologiche, Fondazione Policlinico Universitario A. Gemelli IRCCS, Rome, Italy; 5 Anesthesia and Intensive Care Unit, Department of Emergency and Critical Care, Santa Maria Nuova Hospital, Florence, Italy

**Keywords:** Invasive candidiasis, Biomarker, (1, 3)-β-d-glucan, Downslope

## Abstract

**Background:**

To determine whether a decrease in serum (1,3)-β-d-glucan (BDG) was associated with reduced mortality and to investigate the performance of BDG downslope in predicting clinical outcome in invasive candidiasis.

**Methods:**

Observational cohort study in ICU patients over a ten-year period (2012–2022) in Italy. Proven invasive candidiasis with at least 2 BDG determinations were considered.

**Results:**

In the study population of 103 patients (age 47 [35–62] years, SAPS II score 67 [52–77]) 68 bloodstream and 35 intrabdominal infections were recorded. Serial measurements showed that in 54 patients BDG decreased over time (BDG downslope group) while in 49 did not (N-BDG downslope group). *Candida albicans* was the pathogen most frequently isolated (61%) followed by *C. parapsilosis* (17%) and *C. glabrata* (12%), in absence of any inter-group difference. Invasive candidiasis related mortality was lower in BDG downslope than in N-BDG downslope group (17% vs 53%, *p* < 0.01). The multivariate Cox regression analysis showed the association of septic shock at infection occurrence and chronic liver disease with invasive candidiasis mortality (HR [95% CI] 3.24 [1.25–8.44] *p* = 0.02 and 7.27 [2.33–22.66] *p* < 0.01, respectively) while a BDG downslope was the only predictor of survival (HR [95% CI] 0.19 [0.09–0.43] *p* < 0.01). The area under the receiver operator characteristic curve for the performance of BDG downslope as predictor of good clinical outcome was 0.74 (*p* = 0.02) and our model showed that a BDG downslope > 70% predicted survival with both specificity and positive predictive value of 100%.

**Conclusions:**

A decrease in serum BDG was associated with reduced mortality and a steep downslope predicted survival with high specificity in invasive candidiasis.

**Supplementary Information:**

The online version contains supplementary material available at 10.1186/s13054-024-05022-x.

## Article highlights

- Serum BDG kinetics may correlate with infection course in invasive candidiasis.
- A downslope of serum BDG over time was associated with reduced invasive candidiasis mortality.
- High cutoff levels of BDG downslope predicted survival with high specificity.

**Supplementary Information:**

The online version contains supplementary material available at 10.1186/s13054-024-05022-x.

## Background

Invasive candidiasis (IC) remains the most common serious fungal infection in intensive care unit (ICU) patients and it is still associated with high mortality rates [1]. Prompt diagnosis and appropriate therapy are of upmost importance in this setting; however, in almost half cases blood/tissue cultures could result negative and the identification of pathogens as well as their antifungal susceptibility definition could take some days [2]. As a result, reproducible nonculture-based biomarkers of infection have been widely investigated and approved in clinical practice [3]: among them, (1,3)-β-d-glucan (BDG), a fungal cell wall component of *Candida* spp and other pathogenic fungi [4] can be dosed on patients’ blood sample with reproducible and validated techniques [5]. Its high negative predictive value makes BDG a suitable surrogate marker to support the early diagnosis of several invasive fungal infections, to shorten the time to proper treatment as well as the duration of empirical antifungals therapies, particularly in the ICU setting [6, 7]. Recently, raising interest has been moved towards the prognostic role of BDG and higher baseline values have been associated with worse clinical outcomes [8]. Even though some data in selected population suggested that a decreasing BDG serum level could be related with response to therapies [9], the prognostic role of repeated measurements over time has not been defined yet. We sought to evaluate whether a decreasing trend of serum BDG was associated with lower mortality and to investigate the goodness of BDG downslope cutoff values in predicting clinical outcome, in critically ill patients affected by proven invasive candidiasis.

## Methods

### Patients and setting

We conducted a retrospective analysis of prospectively collected data in consecutive adult patients admitted to the general ICU of an Italian tertiary university hospital, Fondazione Policlinico Universitario A. Gemelli IRCCS (Rome), over a 10-year period between February 2012 and February 2022. Eligibility criteria were as follows: microbiologically confirmed IC, at least 2 serum BDG determinations within the ICU stay, availability of complete clinical and microbiological data. Patients received treatments according to standard local practice and current guidelines [10–12] and, given the study design, microbiological samples including BDG determinations were collected on clinical indication. The observation period for each patient lasted from hospital admission to discharge. The study received approval from the local ethic committee (UCSC1123/11); patients’ informed consent was waived due to the observational design.

### Endpoints

The primary endpoint was to define whether a downslope of serum BDG values over time was associated with a reduced invasive candidiasis related mortality. Secondary endpoints included the investigation of the goodness of BDG downslope cutoff values in predicting survival.

### Variables and measures

Data were acquired from electronic ICU charts (Digistat®) and computerized investigation of microbiology laboratory tests and recorded on an electronic database. These data included: demographic characteristics; medical history and comorbidities—among them, immunosuppressive status was defined by neutropenia (absolute neutrophil count < 500 cells/μL at ICU admission) and/or active neoplasm and/or chronic therapy with steroids or other immunomodulant drugs -; the simplified acute physiology score II (SAPS II) [13] and sequential organ failure assessment (SOFA) score [14]; clinical variables including the source of infection; serum BDG determinations; clinical/technical factors potentially interfering with BDG measurement (e.g., major surgery and surgical gauzes, continuous renal replacement therapy) [15, 16]; microbiological data including pathogens specie identification; antifungal therapy timing, appropriateness and duration; outcome variables namely invasive candidiasis outcome, ICU and hospital mortality, ICU and hospital length of stay.

### Microbiologic methodology and definitions

We considered the first episode of proven IC in each patient when at least two BDG determinations were available. Proven IC was defined by: i) histological evidence of yeast cells or hyphae or pseudo-hyphae from normally sterile site, ii) positive culture for *Candida* species of a sample obtained by a sterile procedure from a normally sterile site, along with clinical manifestations and/or radiological abnormalities consistent with an infectious disease process; iii) candidemia, defined as the isolation of *Candida* species from at least one blood culture from peripheral vein in a patient with consistent clinical manifestations [12, 17]. Both primary and intravenous catheter related bloodstream infections were considered. Catheter related candidemia was defined by a positive catheter tip culture along with isolation of the same *Candida* specie from at least one peripheral vein blood sample culture or by a positive differential time to positivity namely if the blood drawn through the catheter hub yielded positive results at least 120 min earlier than cultures of blood sample drawn from the peripheral vein [18]. Deep-seated infections with secondary candidemia were classified as i) or ii) only. As in our clinical practice, in presence of clinical suspicion of sepsis, at least two blood culture sets consisting of an aerobic and anaerobic bottle (with a blood volume of 16 ml for each set, 8 ml per bottle) were collected and processed using a Bactec (BD Diagnostic Systems, Sparks, MD) or BacT/Alert (bioMérieux, Marcy l’Etoile, France) system. Yeast organisms were isolated after growth on Candida bromcresol green agar plates (Vacutest Kima S.r.l., Arzergrande, Italy) and identified to the species level by matrix-assisted laser desorption ionization–time of flight (MALDI-TOF) mass spectrometry [19]. All positive samples were reviewed by experienced intensivists and an infectious disease specialist.

Antifungal susceptibility testing was performed as part of routine patient care using the Sensititre™ YeastOne ITAMYUCC, (Thermo Fisher Scientific, Waltham, MA, USA) colorimetric plate, by which minimum inhibitory concentration endpoints were visually determined and interpreted according to the current Clinical and Laboratory Standards Institute breakpoints/epidemiological cut-off values to assign susceptibility (or the wild-type phenotype) [20].

The initial antifungal therapy was classified as inappropriate if not including any agent displaying in vitro activity against the isolated pathogen/pathogens. Regarding the class of antifungal drugs and therapy duration, the appropriate treatment was only considered. Source control included surgery, percutaneous procedures, removal of devices and any other intervention in an aim of sterilizing the site of infection. Invasive candidiasis related mortality was defined as death occurring in patients with non-resolving clinical manifestations and still receiving antifungal treatment.

### (1,3) β-d-Glucan assessment

Serum recovered from the patients’ blood samples was tested for BDG according to the manufacturer’s instructions (Fungitell®; Associates of Cape Cod Inc., Falmouth, MA, USA). All BDG tests had a turnaround time ≤ 24 h. All samples were analyzed in duplicate and the mean was assigned as the final result for the specimen; the concentration of BDG in each sample was automatically calculated using a calibration curve with standard solutions and 80 pg/mL was considered the cutoff for test positivity, as recommended [15]. As in our clinical practice, seriate serum BDG tests were performed at intervals of at least 72 h in each patient. BDG downslope was defined as decreasing serum values over time.

### Statistics

The Kolmogorov–Smirnov test was used to evaluate the distribution of data. Variables with a non-normal distribution were expressed as median and selected centile (25–75th) and compared by Mann–Whitney test. Categorical variables were given as proportions and analyzed with the chi-square test or Fisher’s exact test, as appropriate. A descriptive analysis was performed categorizing patients according to the presence or not of a downslope of BDG values over time. Cox regression models of variables associated with IC related mortality were achieved. In the multivariate regression analysis, we considered clinically relevant variables that reached *p* ≤ 0.1 on the univariate analysis and a stepwise selection procedure was used to include variables in the final model. The Kaplan–Meier method was used for the survival analysis. Patients showing a downslope of BDG were categorized in 10 groups according to growing cutoff values of percentage difference between first and last BDG determinations. Sensitivity, specificity, positive predictive value (PPV) and negative predictive value (NPV) of BDG slope cutoff values were computed with standard methods. A receiver operating characteristic (ROC) curves was plotted for an overall assessment of the discriminant power of the BDG slope. All statistical analyses were performed by SPSS version 28 (IBM Software).

## Results

### Population characteristics

In the study period we enrolled 103 patients with median age and SAPS II score of 67 [52–77] years and 47 [35–62], respectively (Table 1). Sixty-eight candidemiae and 35 intrabdominal infections occurred; among bloodstream infections, 18 (17%) events were catheter related. In 54 patients serum BDG values decreased over time (BDG downslope group) while in 49 did not (N-BDG downslope group). We did not record between-group differences in demographics and comorbidities, except for the incidence of chronic renal failure which was higher in patients of the N-BDG downslope than BDG downslope group: 35% vs 15%, *p* = 0.02. An immunosuppressive status was recorded in 43 (42%) patients, with similar rate in both groups (*p* = 0.34). Candidemia occurred more frequently in N-BDG downslope than BDG downslope group, 78% vs 56%, *p* = 0.02. Conversely, intrabdominal infections were recorded with higher rate in BDG downslope rather than N-BDG downslope patients, 44% vs 22%, *p* = 0.02. At the time of infection, the overall median SOFA score was 8 [5–11] and 65% of patients presented with septic shock, without any between-group difference (*p* = 0.08 and *p* = 0.41, respectively). The overall initial BDG value was 500 [254–587] pg/ml and at the end of treatment it was significantly lower in patients of the BDG downslope than N-BDG downslope group: 132 [80–296] vs 500 [426–566] pg/ml, *p* < 0.01. The rate of BDG values > 500 pg/ml was similar between groups at inclusion (*p* = 0.33) whilst it was higher in the N-BDG downslope than BDG downslope group at the end of treatment (71% vs 11%, *p* < 0.01). An overall median of 3 BDG determinations was collected in each patient, without inter-group differences (*p* = 0.29). *Candida albicans* was the pathogen most frequently isolated in both groups, followed by *C. parapsilosis* and *C. glabrata* (overall incidence of 61%, 17% and 12%, respectively); seven patients (7%) had poli-fungal infections. Echinocandins (63%), azoles (20%) and amphotericin B (17%) were administered with similar rate in both groups (*p* = 0.57, *p* = 0.40 and *p* = 0.52, respectively). The antifungal treatment lasted for an overall median of 13 [7–20] days, in absence of inter-group differences (*p* = 0.30). In 52 (50%) patients source control interventions were performed, with similar rate in both groups (*p* = 0.17). Risk and/or confounding factors for BDG serum determination did not differ between groups (ESM, Table S1).

**Table 1:** Clinical characteristics of the 103 patients included in the study

Variable	No. (%) of patients			*p* value
	Total population (n = 103)	BDG downslope (*n* = 54)	N-BDG downslope (*n* = 49)	
*Demographics and comorbidities*				
Age [IQR], years	67 [52–77]	67 [52–79]	67 [51–74]	0.60
Males	62 (60)	32 (59)	30 (61)	1.00
Medical admission	64 (62)	34 (63)	30 (61)	1.00
Surgical admission	31(30)	18 (33)	13 (27)	0.50
Trauma admission	8 (8)	2 (4)	6 (12)	0.15
Chronic heart failure	17 (17)	5 (9)	12 (24)	0.06
Chronic obstructive pulmonary disease	26 (25)	10 (19)	16 (33)	0.12
Chronic renal failure	25 (24)	8 (15)	17 (35)	**0.02**
Diabetes	25 (24)	10 (19)	15 (31)	0.17
Chronic liver disease	6 (6)	3 (6)	3 (6)	1.00
Immunosuppressive status	43 (42)	21 (39)	22 (45)	0.34
SAPS II score at ICU admission [IQR]	47 [35–62]	45 [30–57]	51 [41–63]	0.08
*Presenting features*				
Candidemia	68 (66)	30 (56)	38 (78)	**0.02**
* Catheter-related candidemia*	*18 (17)*	*7 (13)*	*11 (22)*	*0.30*
Intrabdominal infection	35 (34)	24 (44)	11 (22)	**0.02**
SOFA score at infection [IQR]	8 [5–11]	7 [4–10]	9 [6–11]	0.08
Septic shock at occurrence of infection	67 (65)	33 (61)	34 (69)	0.41
ICU stay before infection [IQR], days	2 [0–14]	2 [0–14]	1 [0–20]	0.99
*Microbiologic features*				
Initial BDG [IQR], pg/ml	500 [254–587]	409 [253–720]	500 [275–500]	0.90
Initial BDG > 500 pg/ml	51 (50)	24 (44)	27 (55)	0.33
End-of-treatment BDG [IQR], pg/ml	296 [121–500]	132 [80–296]	500 [426–566]	** < 0.01**
End-of-treatment BDG > 500 pg/ml	41 (40)	6 (11)	35 (71)	** < 0.01**
Number of BDG determinations [IQR]	3 [2–5]	4 [2–6]	3 [2–5]	0.29
*C. albicans*	63 (61)	35 (65)	28 (57)	0.54
*C. krusei*	4 (4)	1 (2)	3 (6)	0.34
*C. glabrata*	12 (12)	6 (11)	6 (12)	1.00
*C. tropicalis*	11 (11)	8 (15)	3 (6)	0.20
*C. parapsilosis*	17 (17)	7 (13)	10 (20)	0.43
*C. dublinensis*	3 (3)	1 (2)	2 (4)	0.60
More than one *Candida* spp	7 (7)	5 (9)	2 (4)	0.27
*Therapeutic aspects*				
Time from BDG determination to treatment [IQR], hours	12 [0–42]	7 [0–24]	24 [0–48]	0.69
Initial inappropriate antifungal therapy	43 (42)	20 (37)	23 (47)	0.33
Azoles	21 (20)	10 (19)	11 (22)	0.40
Echinocandins	65 (63)	33 (61)	32 (65)	0.57
Amphotericin B	17 (17)	9 (17)	8 (16)	0.52
Duration of antifungal therapy [IQR], days	13 [7–20]	14 [8–21]	13 [5–20]	0.30
Source control interventions	52 (50)	31 (57)	21 (43)	0.17
*Clinical and microbiological outcomes*				
Invasive candidiasis related mortality	35 (34)	9 (17)	26 (53)	** < 0.01**
ICU mortality	54 (52)	23 (43)	31 (63)	**0.04**
Hospital mortality	66 (64)	30 (56)	36 (73)	0.05
ICU length of stay after infection [IQR], days	14 [6–26]	18 [8–30]	12 [5–19]	**0.01**
Hospital length of stay after infection [IQR], days	27 [10–52]	35 [13–66]	19 [6–40]	** < 0.01**

Bold value represents *p* < 0.05

Data are shown as N (%), unless otherwise indicated

*BDG* (1,3)-β-d-glucan, *IQR* interquartile range, *SAPS II* simplified acute physiology score, *ICU* intensive care unit, *SOFA* sequential organ failure assessment

### Invasive candidiasis related mortality and other clinical outcomes

**Fig. 1:**
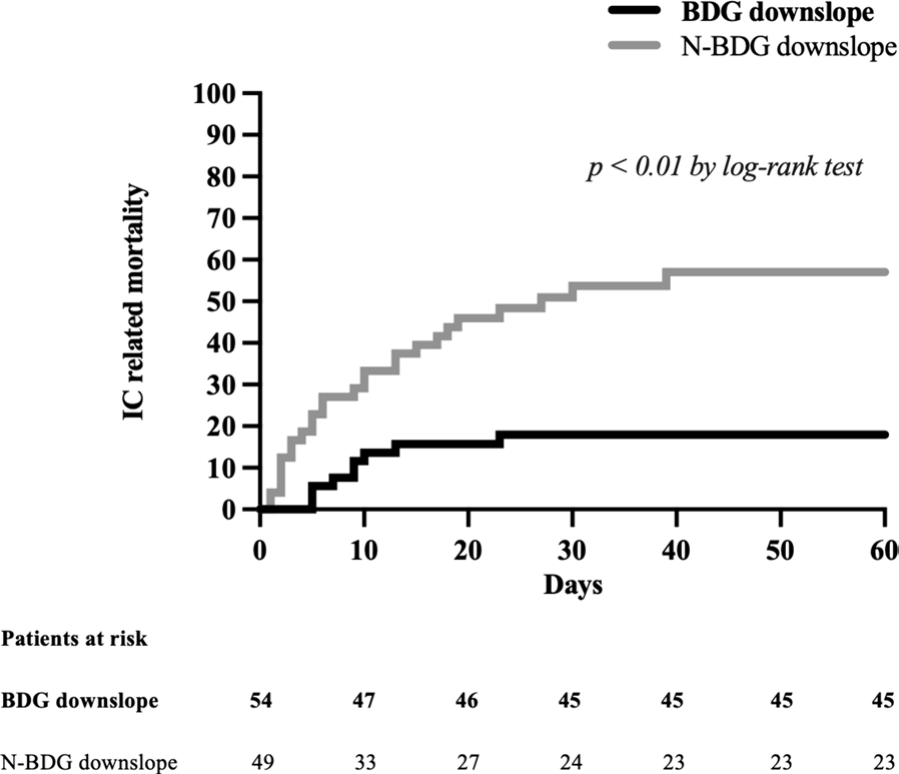
Kaplan–Meier curves showing the cumulative incidence of invasive candidiasis related mortality in patients of the BDG and N-BDG downslope groups. BDG, (1,3)-β-d-glucan; IC, invasive candidiasis

In the BDG downslope group invasive candidiasis mortality was lower (17% vs 53%, *p* < 0.01) as compared to N-BDG downslope group; in more than half patients of both groups IC related mortality occurred within 10 days from infection (Fig. 1). Intensive care unit mortality was lower (43% vs 63%, *p* = 0.04) while ICU and hospital lengths of stay were longer (18 [8–30] vs 12 [5–19] days, p = 0.01 and 35 [13–66] vs 19 [6–40] days, *p* < 0.01, respectively) in BDG downslope than N-BDG downslope group. At the univariate Cox regression analysis the variables associated with IC mortality were: chronic liver disease (HR [95% CI] 3.04 [1.06–8.70], *p* = 0.04), septic shock at infection (HR [95% CI] 3.45 [1.34–8.90], *p* = 0.01), initial BDG > 500 pg/ml (HR [95% CI] 2.38 [1.19–7.75], *p* = 0.01) and BDG downslope (HR [95% CI] 0.23 [0.11–0.49], *p* < 0.01). The multivariate regression analysis confirmed the association of septic shock at infection occurrence and chronic liver disease with invasive candidiasis related mortality (HR [95% CI] 3.24 [1.25–8.44] *p* = 0.02 and 7.27 [2.33–22.66] *p* < 0.01, respectively); conversely a BDG downslope was the only predictor of survival (HR [95% CI] 0.19 [0.09–0.43] *p* < 0.01—Table 2). Factors potentially interfering with serum BDG determinations showed no association with IC mortality (ESM, Table S2).

**Table 2:** Cox regression analysis of factors associated with invasive candidiasis related mortality

Variable	No. (%) of patients		Univariate analysis		Multivariate analysis	
	Alive (*n* = 68)	Deceased (*n* = 35)	HR (95% CI)	*p* value	HR (95% CI)	*p* value
*Demographics and comorbidities*						
Age [IQR], years	64 [46–77]	70 [60–78]	1.01 [0.99–1.03]	0.57		
Males	40 (59)	22 (63)	0.79 [0.39–1.59]	0.51		
Medical admission	42 (62)	22 (63)	1.19 [0.59–2.38]	0.63		
Surgical admission	20 (29)	11 (31)	0.96 [0.46–1.98]	0.91		
Trauma admission	5 (7)	3 (9)	1.03 [0.32–3.39]	0.96		
Chronic heart failure	8 (12)	9 (26)	1.82 [0.85–3.91]	0.12		
Chronic obstructive pulmonary disease	16 (24)	10 (29)	1.13 [0.54–2.36]	0.74		
Chronic renal failure	14 (21)	11 (31)	1.23 [0.60–2.53]	0.58		
Diabetes	16 (24)	9 (26)	0.92 [0.43–1.96]	0.82		
Chronic liver disease	2 (3)	4 (11)	3.04 [1.06–8.70]	**0.04**	7.27 [2.33–22.66]	** < 0.01**
Immunosuppressive status	29 (43)	14 (40)	1.15 [0.59–2.27]	0.68		
SAPS II score at ICU admission [IQR]	45 [30–61]	51 [41–63]	1.02 [0.99–1.03]	**0.10**	1.01 [0.99–1.03]	0.21
*Presenting features*						
Candidemia	43 (63)	25 (71)	1.72 [0.82–3.61]	0.15		
*Catheter related candidemia*	*12 (18)*	*6 (17)*	*0.94 [0.39–2.28]*	*0.90*		
Intrabdominal infection	25 (37)	10 (29)	0.58 [0.28–1.22]	0.15		
SOFA score at infection [IQR]	7 [4–9]	10 [7–12]	1.02 [0.99–1.05]	0.23		
Septic shock at occurrence of infection	37 (54)	30 (86)	3.45 [1.34–8.90]	**0.01**	3.24 [1.25–8.44]	**0.02**
*Microbiologic features*						
Initial BDG [IQR], pg/ml	379 [216–610]	500 [368–500]	1.04 [0.98–1.03]	0.87		
Initial BDG > 500 pg/ml	29 (43)	22 (63)	2.38 [1.19–7.75]	**0.01**		
BDG downslope	45 (66)	9 (26)	0.23 [0.11–0.49]	** < 0.01**	0.19 [0.09–0.43]	** < 0.01**
*C. albicans*	45 (66)	18 (51)	0.56 [0.28–1.11]	**0.10**	0.71 [0.35–1.45]	0.35
Non-*C. albicans*	23 (34)	17 (49)	1.78 [0.90–3.52]	**0.10**		
More than one *Candida* spp	7 (11)	0 (0)	0.09 [0.02–3.07]	0.15		
*Therapeutic aspects and outcomes*						
Time from diagnosis to treatment [IQR], hours	18 [0–48]	0 [0–24]	0.99 [0.98–1.01]	0.25		
Initial inappropriate antifungal therapy	31 (46)	12 (34)	0.65 [0.32–1.31]	0.23		
Azoles	17 (25)	4 (11)	0.62 [0.22–1.76]	0.37		
Echinocandins	40 (59)	25 (71)	1.49 [0.71–3.10]	0.29		
Amphotericin B	9 (13)	8 (23)	1.08 [0.47–2.49]	0.86		
Source control interventions	36 (53)	16 (46)	0.61 [0.31–1.20]	0.15		

Bold value represents *p* < 0.05

Data are shown as N (%), unless otherwise indicated

*HR* hazard ratio, *CI* confidence interval, *IQR* interquartile range, *SAPS II* simplified Acute Physiology Score, *ICU* intensive care unit, *SOFA* sequential organ failure assessment, *BDG* (1,3)-β-d-glucan

### Performance of BDG downslope as a predictor of survival

**Fig. 2:**
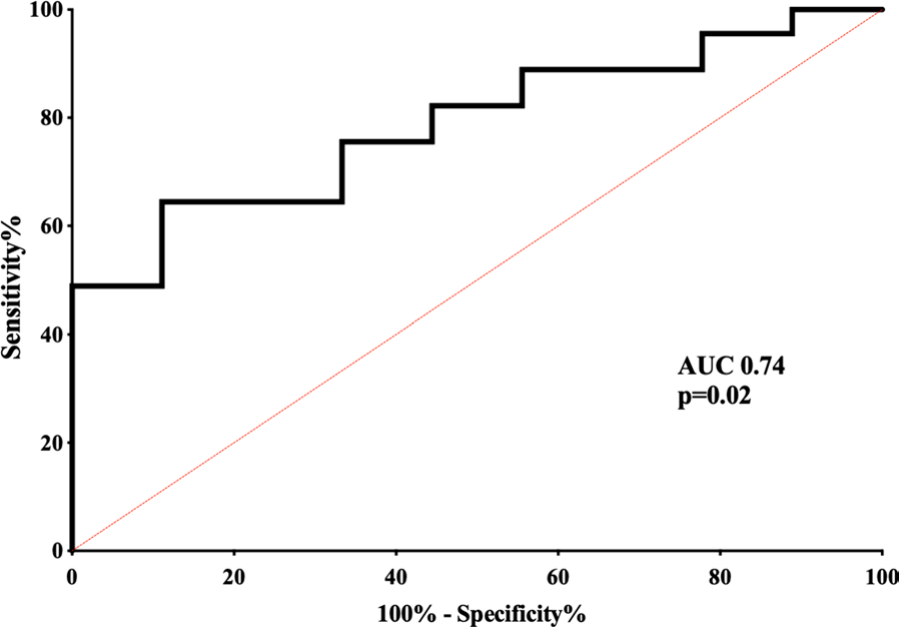
Receiver operator characteristic curve of (1,3)-β-d-glucan downslope cutoff values to define invasive candidiasis related clinical outcome. The slope is expressed as 10-grouped variation in percentage between first and last (1,3)-β-d-glucan values (cfr. Table 3 for details)

We observed growing specificity and positive predictive value as well as decreasing sensitivity in predicting IC related survival at higher cutoff percentage values of BDG downslope (Table 3). An overtime reduction > 50% of the initial BDG value predicted survival with a specificity of 67%, a positive predictive value of 91% and a sensitivity of 64%. In case of a BDG downslope > 70% both specificity and positive predictive value for survival reached 100%. The area under the receiver operator characteristic curve for the performance of BDG downslope as predictor of survival was 0.74 (*p* = 0.02) (Fig. 2).

**Table 3:** Performance of (1,3)-β-d-glucan downslope as a predictor of invasive candidiasis related survival at grouped cutoff levels

Group	BDG variation (%)	TP (downslope and survival)	FP (downslope and death)	TN (non-downslope and death)	FN (non-downslope and survival)	Sen (%)	Spe (%)	PPV (%)	NPV (%)
1	> 0	45	9	0	0	100	0	83	–
2	> 10	43	8	1	2	96	11	84	33
3	> 20	40	7	2	5	89	22	85	29
4	> 30	37	5	4	8	82	44	88	33
5	> 40	34	4	5	11	76	56	89	31
6	> 50	29	3	6	16	64	67	91	27
7	> 60	22	1	8	23	49	89	96	26
8	> 70	14	0	9	31	31	100	100	23
9	> 80	8	0	9	37	18	100	100	20
10	> 90	4	0	9	41	9	100	100	18

Patients in whom a BDG downslope was recorded are only considered (n = 54). The “downslope/non-downslope” indicated in brackets in the columns head refer to the amount of BDG downslope in different rows-cutoffs

The slope is expressed as variation in percentage between first and last (1,3)-β-d-glucan values

*BDG* (1,3)-β-d-glucan, *TP* true positive, *FP* false positive, *TN* true negative, *FN* false negative, *Sen* sensitivity, *Spe* specificity, *PPV* positive predictive value, *NPV* negative predictive value

## Discussion

In the study, a downslope of BDG values was independently associated with reduced invasive candidiasis related mortality and performed as a good predictor of clinical outcome, with high survival rate in patients with wider BDG reduction.

The hypothesis of a relation between BDG kinetics and clinical outcomes lies on the assumption that a predicted model of BDG serum levels could be expected in successfully treated infections: an initial zenith due to the disruption of the cell wall is followed by a reduction, as yeast cells are no longer reproducing and BDG is cleared. A recent retrospective study on candidemic patients showed that those with persistently negative BDG determinations had better clinical outcomes, probably due to a lower hematogenic fungal inoculum [21]. However, several factors could interfere with this theoretical model limiting its clinical applicability in invasive fungal infections: the source and burden of infection; the type of antifungals administered: polyenes and echinocandins are fungicidal and they are expected to cause major spread and hence higher initial level of BDG as compared to azoles that are fungistatic; similarly, drugs dosage and bioavailability depending on concomitant clinical conditions and treatments (e.g., septic shock, continuous renal replacement therapies); the renal clearance; other confounding factors (e.g., external sources of BDG such as surgical gauzes, hemodialysis, etc.) [16, 22].

In vivo, the prognostic role of the BDG kinetics has been investigated in patients with invasive fungal infections such as aspergillosis and pneumocystis jirovecii pneumonia (PJP). In these study populations, authors found associations between response to therapies and marker downslope on one side and between treatment failure and marker increase on the other side, both in animal models and human subjects [23–26]. Conversely, other authors found a normalization of BDG values after effective antifungal treatment only in a minority of PJP patients [27, 28], although including mainly neutropenic patients where the microbiological eradication could have followed the clinical cure. To date, few and heterogeneous data are available dealing with BDG prognostic role in *Candida* infections. In a population of patients affected by IC, Jaijakul et al. [9] observed that a reduction of BDG over time was associated with positive response to treatment. However, this study was not focused on the critically ill setting, the deep majority of patients had candidemia due to *non-C. albicans* pathogens and the only antifungal administered was anidulafungin. Sims et al. [29] conducted a prospective observational study on BDG trend in a population of patients, including critically ills, with proven IC mainly in form of candidemia: even though in absence of significant changes, they found that BDG tent to decrease in successfully treated patients and to increase in those who did not present a clinical response to treatments. More recently, Träger et al. [30] observed a reduction of serum BDG and mannan levels in successfully treated patients with candidemia; conversely, an increase of these markers was associated with the persistence/recurrence of the bloodstream infection. Our observations confirmed and broadened these previous findings as the association between BDG downslope and better clinical outcome was documented, in a population of ICU patients with heterogeneous invasive candidiasis treated with different antifungals. Moreover, unlike in previous studies, both candidiasis and deep-seated infections were well represented in our population and showed different incidence in BDG and N-BDG downslope groups. Interestingly, we observed that patients in the BDG downslope group had a higher rate of intrabdominal infections whilst the N-BDG downslope group was mainly affected by candidemia: this finding could suggest that a “local” infection could have brought a lower systemic BDG burst also due to the possibility of effective interventional infection control, hence enhancing a better clearance of both the infection and the BDG. However, we observed no differences in the rate of source control interventions between groups, indicating that they were not sufficient to determine a BDG downslope. Consistently, in a previous study, central venous catheter removal was observed not to be related with BDG variations as compared with device maintenance in patients with proven catheter-related candidemia [29]. The pattern factors potentially interfering with serum BDG levels were also similar in our study groups suggesting no main impact of these underlying conditions on the marker slope.

In an attempt of evaluating the goodness of the marker as a predictor of clinical outcome, we categorized patients of the BDG downslope group according with the percentage of reduction between the first and last serum values. We observed that the higher was the BDG downslope cutoff, the better were its specificity and positive predictive value for IC survival, while sensitivity and negative predictive value showed an inverse trend. Hence, when a steep BDG downslope was detected, the probability of survival increased progressively, reaching 100% in case of a reduction > 70% from the initial value. To our knowledge, such a correlation has never been explored. In a retrospective analysis, Giacobbe et al. [31] investigated the prognostic role of the initial value of BDG in candidemic patients and found that a > 287 pg/mL cutoff predicted 28-day mortality with the best sensitivity and specificity. Pini et al. [32] recorded data on a population of patients with proven and probable invasive fungal infections and determined 0.6263 pg/ml/day as the cutoff value of daily BDG downslope able to predict the clinical outcome with the best performance. If further and larger prospective investigations could confirm the goodness of BDG downslope values to predict survival in invasive candidiasis, this finding could assume a relevant clinical usefulness.

The multivariate Cox regression analysis also confirmed the association of septic shock at the occurrence of infection and chronic liver disease with IC related mortality. Septic shock is the most severe clinical presentation of IC and it is well known to be associated with poor clinical outcomes [33]. Patients with cirrhosis are at increased risk of invasive fungal infections and among them the most frequent is candidiasis, particularly in the ICU setting and in presence of other risk factors (e.g., abdominal surgery, prolonged ICU stay, acute kidney injury and renal replacement therapies) [34]. Invasive infections have been associated with higher mortality rates in cirrhotic rather than in non-cirrhotic patients [35], even though few data are available regarding fungal infections specifically, some reporting similar mortality rate as compared to other populations [36]. Nevertheless, the overall small number of patients presenting this comorbidity in our study prevented us to draw conclusions about this peculiar issue.

The findings of the present study could improve the use of BDG as a marker of infection control and clinical course. A BDG downslope, particularly in presence of marked reduction, could suggest good clinical outcome and response to ongoing treatments; conversely, in absence of BDG downslope further source control interventions and/or antifungal therapies changes might be considered.

We are aware of some limitations of our study. First, given the monocentric setting and the observational design, the reproducibility of our results is limited. Second, the number and timing of BDG determinations were only driven by clinical practice and hence not standardized. Third, recent exposure to antifungals was not recorded and it could had influenced both the initial value and the kinetics of BDG. Finally, the lack of control over antifungals usage could have affected our findings; in addition, due to the limited sample size we could not perform subgroup analysis focusing on different drug classes effects.

## Conclusions

Serum BDG downslope was associated with reduced mortality and a steep decrease turned out to be a good predictor of clinical outcomes in invasive candidiasis. The definition of a prognostic role for the trend of BDG values over time could encourage a personalized clinical approach with the aim at improving outcomes in critically ill patients with invasive candidiasis.

### Supplementary Information

**Additional file 1**.

## Data Availability

The datasets used and/or analyzed during the current study are available from the corresponding author under reasonable request.

## Abbreviations

BDG: (1, 3)-β-D-glucan
IC: Invasive candidiasis
ICU: Intensive care unit
SAPS II: Simplified acute physiology score II
SOFA: Sequential organ failure assessment
ESM: Electronic supplementary material
MALDI-TOF: Matrix-assisted laser desorption ionization–time of flight
HR: Hazard ratio
CI: Confidence interval
PPV: Positive predictive value
NPV: Negative predictive value
ROC: Receiver operating characteristic
