# Supplementary material for: Prognostic value of serial (1,3)-β-d-glucan measurements in ICU patients with invasive candidiasis
Source: Crit Care. 2024 Jul 12;28:236. doi: 10.1186/s13054-024-05022-x (PMC11241937; doi:10.1186/s13054-024-05022-x)
Supplement: Supplementary file 1 — Additional file 1. [file 13054_2024_5022_MOESM1_ESM.docx]

**Electronic supplementary material.**

**Table S1.** Risk/confounding factors for serum BDG levels of the 103 patients included in the study.

|  | No. (%) of patients | | | *p* value |
| --- | --- | --- | --- | --- |
| Variable | **Total population** (n=103) | **BDG downslope**  (*n*=54) | **N-BDG downslope**  (*n*=49) |  |
| Previous abdominal/major surgery | 59 (57) | 32 (59) | 27 (55) | 0.69 |
| Parenteral nutrition | 17 (17) | 8 (15) | 9 (18) | 0.79 |
| Continuous renal replacement therapy | 36 (35) | 17 (31) | 19 (39) | 0.54 |
| Treatment with intravenous immunoglobulins | 8 (8) | 4 (7) | 4 (8) | 1.00 |
| Serum albumin [IQR], g/dl | 2.4 [2.1-2.7] | 2.5 [2.1-2.7] | 2.3 [2.0-2.7] | 0.17 |
| Serum bilirubin > 10 mg/dl | 6 (6) | 3 (6) | 3 (6) | 1.00 |
| Treatment with beta-lactams | 86 (83) | 46 (85) | 40 (82) | 0.79 |
| Surgical gauzes | 10 (10) | 3 (6) | 7 (14) | 0.19 |

**Table S2.** Univariate Cox regression analysis of risk/confounding factors for serum BDG levels associated with invasive candidiasis related mortality.

|  | **No. (%) of patients** | | **Univariate analysis** | |
| --- | --- | --- | --- | --- |
| Variable | **Alive**  (*n*=68) | **Deceased**  (*n*=35) | HR (95% CI) | *p* value |
| Previous abdominal/major surgery | 36 (53) | 23 (66) | 1.56 [0.77-3.14] | 0.22 |
| Parenteral nutrition | 11 (16) | 6 (17) | 0.78 [0.33-1.88] | 0.58 |
| Continuous renal replacement therapy | 21 (31) | 15 (43) | 1.03 [0.52-2.04] | 0.94 |
| Treatment with intravenous immunoglobulins | 5 (7) | 3 (9) | 1.42 [0.43-4.67] | 0.57 |
| Serum albumin [IQR], g/dl | 2.4 [2.1-2.6] | 2.3 [2-2.7] | 0.80 [0.40-1.60] | 0.53 |
| Serum bilirubin > 10 mg/dl | 4 (6) | 2 (6) | 0.87 [0.21-3.61] | 0.84 |
| Treatment with beta-lactams | 57 (84) | 29 (83) | 0.92 [0.38-2.22] | 0.85 |
| Surgical gauzes | 5 (7) | 5 (14) | 2.25 [0.86-5.85] | 0.10 |
